# Israeli Acute Paralysis Virus: Honey Bee Queen–Worker Interaction and Potential Virus Transmission Pathways

**DOI:** 10.3390/insects10010009

**Published:** 2019-01-08

**Authors:** Esmaeil Amiri, Gregory Seddon, Wendy Zuluaga Smith, Micheline K. Strand, David R. Tarpy, Olav Rueppell

**Affiliations:** 1 Department of Biology, University of North Carolina at Greensboro, Greensboro, NC 27402-6170, USA; gregvbs@gmail.com (G.S.); zuluagaw1@gmail.com (W.Z.S.); o_ruppel@uncg.edu (O.R.); 2 Department of Entomology & Plant Pathology, North Carolina State University, Raleigh, NC 27695-7613, USA; david_tarpy@ncsu.edu; 3 Life Science Division, U.S. Army Research Office, Research Triangle Park, Durham, NC 27709-2211, USA; micheline.k.strand.civ@mail.mil

**Keywords:** queen, honey bee viruses, Israeli acute paralysis virus, queen loss, virus transmission

## Abstract

Queen loss or failure is an important cause of honey bee colony loss. A functional queen is essential to a colony, and the queen is predicted to be well protected by worker bees and other mechanisms of social immunity. Nevertheless, several honey bee pathogens (including viruses) can infect queens. Here, we report a series of experiments to test how virus infection influences queen–worker interactions and the consequences for virus transmission. We used Israeli acute paralysis virus (IAPV) as an experimental pathogen because it is relevant to bee health but is not omnipresent. Queens were observed spending 50% of their time with healthy workers, 32% with infected workers, and 18% without interaction. However, the overall bias toward healthy workers was not statistically significant, and there was considerable individual to individual variability. We found that physical contact between infected workers and queens leads to high queen infection in some cases, suggesting that IAPV infections also spread through close bodily contact. Across experiments, queens exhibited lower IAPV titers than surrounding workers. Thus, our results indicate that honey bee queens are better protected by individual and social immunity, but this protection is insufficient to prevent IAPV infections completely.

## 1. Introduction

The European honey bee, *Apis mellifera*, lives in colonies containing several thousand, closely related workers typically derived from a single queen. The queen is central to the colony, releasing pheromones and producing all of the eggs that replenish and grow the colony workforce and produce sexuals for the next generation [1,2]. The non-reproductive members of the colony, the worker bees, perform all other colony tasks, including queen care [1]. The queen’s pheromones attract a retinue of worker bees to attend the queen by antennating, grooming, and feeding her, which includes close physical contact and liquid food transfer via trophallaxis [3,4,5]. These retinue behaviors facilitate the spread of the queen pheromones to the rest of the colony, but the worker–queen interactions are also crucial for maintaining queen health and (presumably) responsible for facilitating the exceptional longevity of the queen [6,7,8]. Since retinue workers are typically young with little contact to the outside world, they may provide a social barrier to protect the queen and lower her exposure to infectious diseases [9,10]. Nevertheless, any physical contacts and trophallactic interactions have the potential for horizontal disease transmission to compromise queen health [11,12].

Different pathogens can affect queen health, but honey bee viruses are of particular concern [13]. Several honey bee viruses persist as covert infections in queens of apparently healthy colonies [14,15,16,17]. Among these viruses, deformed wing virus and the acute bee paralysis complex (acute bee paralysis virus, Israeli acute paralysis virus, and Kashmir bee virus) have been specifically associated with declining honey bee health [18,19,20], although the pathogen signature of collapsing colonies is complex [21]. Israeli acute paralysis virus (IAPV) is one of several RNA viruses from the family *Dicistroviridae* [22], and while it has been reported on almost all continents, it is not omnipresent [23]. IAPV can be transmitted within and among honey bee colonies by a combination of horizontal and vertical transmission pathways [24]. It has been found in pollen and bee feces, and it can be actively vectored by *Varroa* mites [24,25]. In naturally infected colonies, IAPV can be detected in all honey bee developmental stages (including eggs), as well as adult workers, queens, and males (drones). It can infect all honey bee tissues, but particularly high levels are found in the gut, nervous system, and hypopharyngeal glands [24]. Workers that are experimentally infected with IAPV display shivering wings, cramping, and disorientation before their paralysis, and death within a few days [26,27]. These pathologies are accompanied by a major disruption of the hosts’ transcriptome [27,28,29].

Honey bees have evolved several individual and colony-level defense mechanisms against pathogens. Like all other animals, individual honey bees use mechanical barriers, physiological and behavioral responses, and a specialized immune system to defend themselves against pathogens [30,31]. However, honey bees exhibit additional adaptations to combat diseases at the group level, which have been termed “social immunity” [32]. Behavioral forms of social immunity include hygienic behavior, allogrooming, altruistic self-removal, and resin collection [33,34,35,36,37]. However, there may also be more subtle mechanisms of social immunity that have not been sufficiently investigated. For example, the centrifugal age-polyethism of workers in many social insects brings only the youngest workers in direct contact with the queen and brood, which may represent a demographic means of social immunity. Social immunity may also interact with individual immunity by social transfers [38,39]. In addition, immune-challenged worker honey bees exhibit modified cuticular hydrocarbon profiles, which increases antennation and grooming by nestmates [40,41]. Few studies of social immunity have explicitly addressed the protection of the queen despite her crucial role in colony function and health, in order to explore the theoretical possibility and tentative evidence for caste-specific protection [13,42].

To address how honey bee queens may be specifically protected from IAPV, and to further understand social immunity, we studied the behavioral interactions between queens and workers then quantified the resulting IAPV transmission from experimentally infected workers to non-infected queens. In a series of three laboratory experiments, we increasingly afforded queens the opportunity to associate with or avoid IAPV-infected workers. We report behavioral and molecular data that suggests the social and individual immunity mechanisms can partially protect honey bee queens from horizontal transmission of viral infections from infected nestmates.

## 2. Materials and Methods

### 2.1. Materials

After a visual inspection and qPCR screening for virus infections (see below), three healthy colonies from the research apiary at the University of North Carolina at Greensboro (UNCG), Greensboro, North Carolina, were selected as sources for all honey bees involved in the experiments. These source colonies were selected based on (1) low infestation levels of *Varroa* mites (≤1 mite in 300 worker bees); (2) absence of overt diseases symptoms (e.g., European foulbrood, American foulbrood, and chalkbrood); and (3) absence of physical symptoms from IAPV and related viruses of the acute bee paralysis complex. One of these source colonies was set up as a queen-less queen-rearing hive, according to standard practices [43].

For each experiment, sister queens were produced by standard grafting of 1st–2nd instar larvae from the healthy colony into artificial queen cups that were then placed into the queen-less nurse colony to develop [43]. Two days before queen emergence, all the completed queen cells were transferred to separate cages in an incubator (34 °C and 65% R.H.) to avoid any antagonistic interactions during emergence [44]. Prior to the emergence of queens, frames of capped, ready-to-emerge worker brood from the healthy donor colonies were also transferred to the incubator to let workers emerge in an isolated environment. The resulting queens and workers were raised and used separately in three experiments during the summer of 2016. During each experiment, two queen larvae and their royal jelly, as well as two newly emerged queens, were tested by qPCR (see below) to confirm the IAPV-free status of the experimental subjects at the beginning of each experiment.

Active IAPV-inoculum was obtained following the established protocol [27], with slight modifications for proper pupae collection [45]. Briefly, existing IAPV [27] was propagated in 30 pupae via injection of 2 µL with a micro-injector (0.1 μL/s infusion flow rate, NanoJet, Chemyx Inc., Stafford, TX, USA), using a standard microliter syringe with a 10 µL cemented needle inserted between the second and third abdominal segments of each pupa. The injected pupae were placed in glass petri dishes containing a double-layer of dry, sterile filter paper. Injected pupae were collected 3 d post-injection, and stored in −80 °C for virus purification by density centrifugation and filtration. IAPV concentration was determined by generating a standard curve from a serial dilution of IAPV cDNA. The IAPV stock inoculum contained 5.24 × 10^9^ copies of IAPV per microliter, and a dilution of 10^−2^ from the stock was prepared to inoculate each experimental worker with approximately 10^8^ IAPV particles in 2 μL.

### 2.2. Experiment 1

In order to test the transmission of IAPV from infected workers to healthy queens, this experiment quantified IAPV titers in queens that were housed together with IAPV-infected workers. Newly emerged workers from a healthy brood frame were caged in sterile Plexiglass™ cages (9.9 cm × 9.9 cm × 10.5 cm, custom made) and supplied ad libitum with sugar candy, irradiated pollen, and water. Nineteen unmated (virgin) sister queens emerged 4 d later, and each was housed with fifteen randomly selected worker bees that were inoculated with IAPV at that time. Each worker was anesthetized on ice to remove the dorsal thoracic hairs with a sterile syringe tip then applied with 2 µL of IAPV-inoculum onto the scutum. Workers were gently placed into the cage for the IAPV to be absorbed while the bees were recovering from anesthetization. After inoculation of all 15 workers and absorption of all inoculum (5–10 min), a single virgin queen was introduced to each cage. The cage was kept in the incubator, and sugar candy and water were provided daily. Observations of disease symptoms and mortality were initiated on Day 3 post-infection. Dead bees were not removed from the cages during the experiment to insure physical contact of the queens with potentially infectious materials. As control, five queens with fifteen healthy worker bees each were kept in a separate incubator. The experiment was terminated 9 d post-infection by freezing and storing each queen and all (dead and alive) workers at −80 °C for subsequent molecular analysis.

### 2.3. Experiment 2

A second experiment was designed to study IAPV transmission from workers to virgin queens through trophallaxis without physical contact between queens and IAPV-infected workers. Eighteen sister queens were produced, and newly emerged workers collected from brood of a healthy UNCG colony as described above. Queens and workers were transferred into an emergence incubator, then fifteen worker bees were inoculated with IAPV on the day of queen emergence and placed into a Plexiglass™ cage. The virgin queen was introduced into her own compartment of the cage so that workers were separated from the queen by a 1.5 mm metal mesh screen for the entire experiment. Worker bees were provided with sugar candy, irradiated pollen, and water for their own consumption. They could also feed the queen through the metal screen. The queen compartment did not contain any food or water, forcing the queens to receive food via trophallaxis from the infected worker bees. Disease symptoms were observed, and dead bees from the cages were removed daily. Newly infected bees were added to the cages to keep the worker population constant until the end of experiment on the 10th day. In another incubator, a control group of five additional queens with fifteen healthy workers each were also kept in cages that were separated by a wire mesh to prevent direct physical contact between queens and workers. All queens were individually collected, and the corresponding worker pools were also collected and stored at −80 °C until further analysis.

### 2.4. Experiment 3

In the final experiment, queen–worker interactions were compared between healthy and IAPV-inoculated groups of workers that were paired with the same queen. Ten queens were produced from a healthy UNCG colony, and upon queen emergence, each queen was introduced into a mating hive with 300–400 worker bees to mate freely. After the start of ovipositioning, mated queens were transferred in the laboratory to the experimental cages (see below). We used mated queens to increase the queens’ attractiveness towards workers, triggering more interactions. Twenty worker bees per mating hive were tested to ensure that no IAPV was transmitted from the workers to the experimental queens during mating time. As described above, a frame of healthy, ready-to-emerge worker brood was transferred to an incubator, and newly emerged workers were collected in group of 15 and either incubated with IAPV or left untreated as controls.

The experimental cages had a central queen compartment without food or water in which the queen was introduced. On different sides, two worker compartments bordered the queen chamber, separated by a circular 1.5 mm metal mesh screen of 1 cm diameter. One side chamber of each cage was populated with 15 IAPV-inoculated workers, and the other side chamber was populated with healthy worker bees, so that the whole arrangement functioned as a dual choice apparatus. Worker bees and queens had the opportunity to contact each other through the wire mesh screen. In contrast to the central queens, both worker groups were provided with sugar candy and water, forcing queens to solicit food from either worker group to survive. On subsequent days, queens were observed for 30 s once every hour, from 8:00 am to 5:00 pm, to record interactions with infected or healthy workers (the data are provided in Supplementary file; Queen Choice). At the same time, the number of worker bees that were congregating in front of the connection hole was recorded (the data are provided in Supplementary file; Attendance Behavior). To compensate for the death of infected workers, new IAPV-inoculated bees were added after 6 d. Dead queens and workers were collected immediately and stored at −80 °C. The experiment was terminated after 13 days, and all remaining live queens and workers were frozen at −80 °C and stored with the previously collected samples until further analysis.

### 2.5. Molecular Assays

Frozen honey bees were homogenized using TissueLyser LT (QIAGEN, Hilden, Germany) for 1 min, followed by a total RNA extraction with an established TRIzol™ (Invitrogen, Carlsbad, CA, USA) protocol [46]. Queens were extracted individually using the entire body, while five workers per sample were pooled without the site of inoculation (thorax) to avoid detecting remnants of the experimental IAPV inoculation instead of active infections. The concentration and purity of the extracted RNA samples were measured using a Nanodrop ND-1000 spectrophotometer (Thermo Scientific, Wilmington, DE, USA), and total RNA concentration was adjusted to 20 ng/µL in molecular grade water (Fisher Scientific, Fair Lawn, NJ, USA).

A two-step quantitative PCR assay was carried out to quantify the IAPV viral loads in the samples. For each sample, cDNA was synthesized using the High Capacity cDNA Reverse-Transcription Kit (Applied Biosystems, Foster City, CA, USA). Ten microliters of the RNA template (20 ng/µL) was added to 10 µL of the provided cDNA master mix, followed by an incubation period as recommended by the manufacturer: 10 min at 25 °C, 120 min at 37 °C, and 5 min at 85 °C. The resulting cDNA solution was then diluted 10-fold in molecular grade water to serve as template in subsequent qPCRs using unlabeled primers and SYBR Green DNA binding dye (Applied Biosystems). Quantitative PCR amplifications were carried out on a StepOnePlus™ cycler (Applied Biosystems) in duplicate, and in a reaction volume of 20 µL with the final primer concentrations of 0.4 µM. A positive control was run in each plate, and RNase-free water was added as template for the “No Target Control” (NTC). A No Reverse Transcriptase (NRT) control served as an additional negative control. The reference gene RPS5 was used as an internal control to confirm the integrity of samples and functionality of assay. Fluorescence measurements were taken at the end of each cycle, followed by a final melt curve dissociation analysis to confirm the specificity of the products. Samples were deemed positive for a target when their melting temperature was within 0.5 °C of the melting temperature of the positive controls. The *C*t values were determined at the same fluorescence threshold (0.1) for all plates, and a *C*t value of 36 or lower was recorded as positive amplification. The primers used in this study for IAPV (5′ CCATGCCTGGCGATTCAC 3′ and 5′ CTGAATAATACTGTGCGTATC 3′) and RPS5 (5′-AATTATTTGGTCGCTGGAATTG 3′ and 5′-TAACGTCCAGCAGAATGTGGTA) had previously been validated to detect the intended targets and are commonly used in honey bees [23,47].

### 2.6. Data Analysis

Virus load in each sample was quantified using absolute quantification, based on our standard curves obtained through serial dilutions of known numbers of amplicons as described before [48]. For the samples in which IAPV was not detected, (i.e., those in which the IAPV amplification curve did not reach the fluorescence threshold in 36 cycles, corresponding to a theoretical detection threshold of 90 IAPV copies), the *C*t value was set to 40 (all raw data are provided in Supplementary file; Experiment 1, Experiment 2, Experiment 3). To improve data compliance with parametric assumptions, statistical tests were performed on transformed raw data, according to $x^{\prime }=log_{10}\left(x+1\right)$. Data analysis and visualization were performed using Microsoft Excel and “R”, version 3.1.3.

## 3. Results

### 3.1. Experiment 1

**Figure 1:**
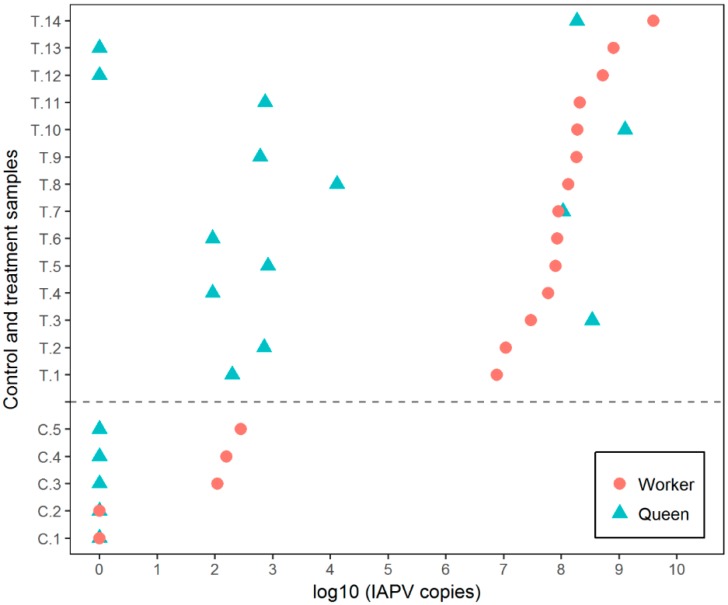
Israeli acute paralysis virus (IAPV) titers of worker and queen samples in control and treatment groups. IAPV-inoculated workers developed virus titers in the range of 7.62 × 10^6^ to 3.91 × 10^9^ copies/µL. Queens in trophallactic and direct physical contact with infected workers became also infected (IAPV titers ranged from 9 × 10^1^ to 1.25 × 10^9^ copies/µL). Two queens with infected workers showed no detectable virus. Queens in the control cages remained uninfected, even though accompanying workers in three experimental cages showed a low level of IAPV (1.09 × 10^2^ to 2.78 × 10^2^ copies/µL). Thus, IAPV titers were lower in queens than in the corresponding workers in 14 cases, while the opposite was true in only 3 cases. T = IAPV-infected workers, C = control workers.

Workers in the experimental cages exhibited disease symptoms after 3 d; they progressed gradually from “nervousness,” to shivering wings and crawling, then death (with considerable inter-individual variability). Dead workers were not removed from the cages during the experiment, therefore, daily mortality was not recorded, but the majority of worker bees were dead by the experimental termination. These overt symptoms were not observed in virgin queens, however, 5 queens died before day 9 post-infection. Virus quantification results confirmed the successful inoculation of the worker bees with IAPV quantities ranging from 7.62 × 10^6^ to 3.91 × 10^9^ copies/µL with an average of 4.49 × 10^8^ copies/µL (Figure 1). IAPV was also detected in all but two queens, with titers ranging from 9 × 10^1^ to 1.25 × 10^9^ copies/µL and an average of 1.35 × 10^8^ copies/µL (Figure 1). All five control queens remained absent of IAPV, even though workers in three of the control cages developed a low level IAPV infection with IAPV titers ranging from 1.09 × 10^2^ to 2.78 × 10^2^ copies/µL (Figure 1). A chi-square test indicates statistical differences between experimental queens in contact with infected workers and the control queens in contact with healthy workers (Fisher’s exact test; *p* = 0.0018). The IAPV titer of queens was not strongly correlated to the IAPV titer of the corresponding workers in the treatment group (*r* = 0.029, *n* = 14, *p* = 0.05). RPS5 was consistently amplified in all samples with an average *C*t values of 20.32 ± 1.75 (SD) and 22.38 ± 1.77 for queens and worker samples.

### 3.2. Experiment 2

**Figure 2:**
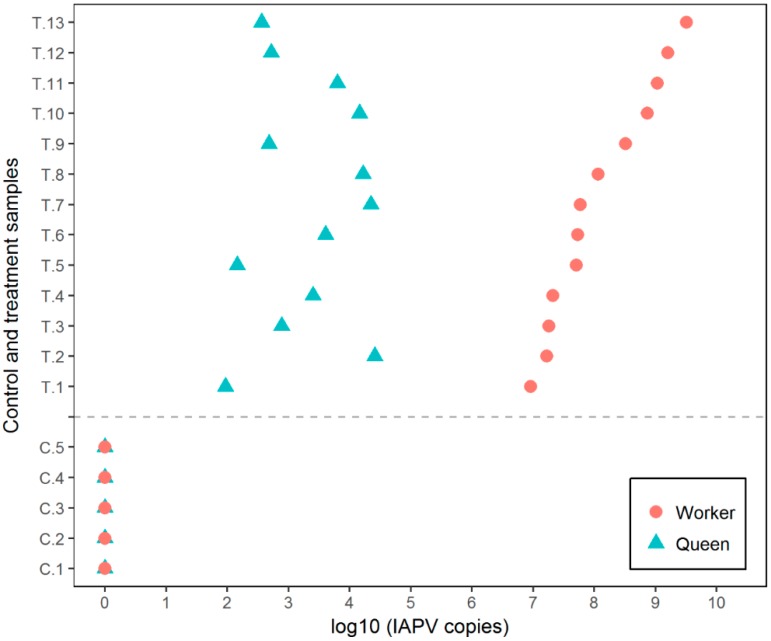
IAPV titers of worker and queen samples in control and treatment groups of trophallactic experiment. IAPV-inoculated workers developed virus to a range from 9.2 × 10^6^ to 3.25 × 10^9^ copies/µL and consequently, queens in trophallactic contact with infected workers become infected (IAPV range from 9.3 × 10^1^ to 2.59 × 10^4^ copies/µL), only due to feeding contact with infectious workers. Queens and workers in the control cages remained uninfected. In all cases with detectable IAPV levels, the titers were higher in workers than in the queens. T = IAPV-infected workers, C = control workers.

IAPV symptoms of workers started 3 d after inoculation, as described above, with the death of majority of workers in each cage by the experimental termination. Daily observation confirmed trophallactic feeding of queens by the worker bees through the metal mesh screen. Trophallactic feeding is known as a common oral–oral virus transmission route, but our experiment was not able to control for any transmission that may have occurred via an airborne route (if any). IAPV titers of infected workers in experimental cages ranged from 9.2 × 10^6^ to 3.25 × 10^9^ copies/µL, with an average of 5.62 × 10^8^ copies/µL (Figure 2). All queens were infected, with IAPV titers ranging from 9.3 × 10^1^ to 2.59 × 10^4^ copies/µL, and an average of 7.28 × 10^3^ copies/µL (Figure 2). Worker and queens in control cages remained absent of IAPV. There was a significant difference between queens in contact with infected workers and the ones in control group (Fisher’s exact test; *p* = 0.0001). Across experimental replicates, the IAPV titer of queens was not positively correlated to the IAPV titer of the corresponding workers (*r* = −0.28, *n* = 13, *p* = 0.05). RPS5 amplified consistently in all queens (*C*t value 20.04 ± 1.8 (SD)) and workers (*C*t value 23.64 ± 1.6).

### 3.3. Experiment 3

**Figure 3:**
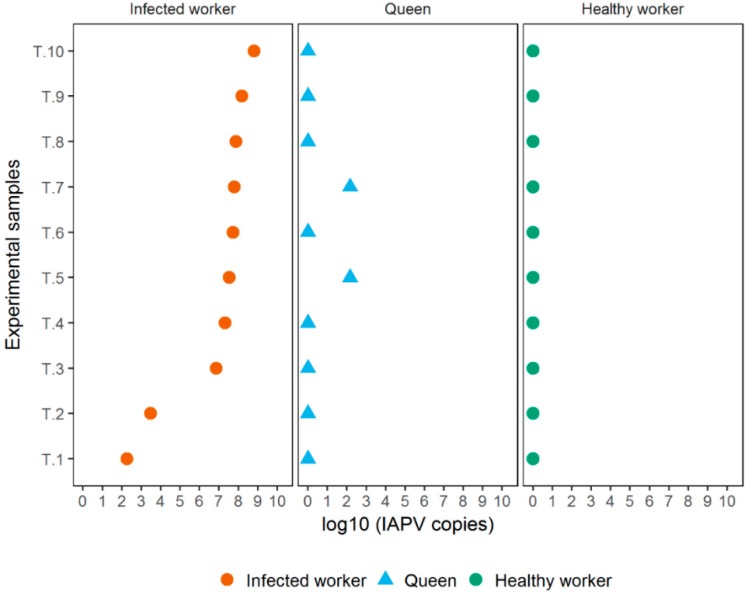
Virus titers of inoculated and non-inoculated workers, and queens in a dual choice experiment. IAPV titers in inoculated workers ranged from 1.84 × 10^2^ to 6.43 × 10^8^ copies/µL, but no virus was detected in the non-inoculated workers. The queens were in trophallactic contact with both worker groups but did not show IAPV-infections, except two with low titers (1.43 × 10^2^ and 1.46 × 10^2^ copies/µL). T1–T10 = independent experimental replicates.

As in the previous experiments, infected workers started to show the symptoms 3 d after inoculation and gradually died, but IAPV symptoms were not seen in non-inoculated workers. IAPV quantification tests showed that virus titers ranged from 1.84 × 10^2^ to 6.43 × 10^8^ copies/µL with an average of 1.05 × 10^8^ copies/µL for the inoculated workers (Figure 3). Healthy workers, on the other side of the queen compartment, were absent of IAPV (Figure 3). No IAPV was detected in the majority of queens, but two exhibited low titers (1.43 × 10^2^ and 1.46 × 10^2^ copies/µL) (Figure 3). RPS5 amplified consistently in the healthy workers (*C*t values: 20.92 ± 1.46), infected workers (*C*t values: 22.48 ± 1.84), and queens (*C*t values: 20.1 ± 2.37).

**Figure 4:**
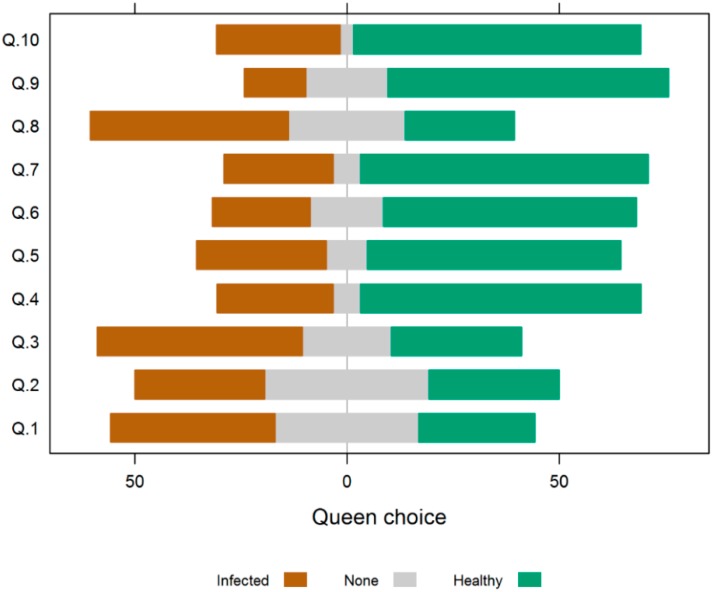
Honey bee queen tendency to associate with healthy or infected workers. For each queen (Q1–Q10), the percentage of time that this queen was in contact with infected workers, healthy workers, or neither group, is indicated by the length of the colored bars. Even though the majority of queens tend to spend more time with healthy workers, the overall preference for healthy workers was not statistically significant (*p* = 0.058), Brown bars to the left = percentage time spent with infected workers, green bars to the right = percentage time spent with healthy workers. The order of independent trials (Q1–Q10) corresponds to the order in Figure 3).

Significant inter-individual variability in queen choice behavior was observed. While more queens preferentially interacted with healthy workers, some did not exhibit a preference, or spent more time in contact with the inoculated workers (Figure 4). Thus, the overall preference for healthy workers was not statistically significant (Wilcoxon signed rank test: *p* = 0.058).

**Figure 5:**
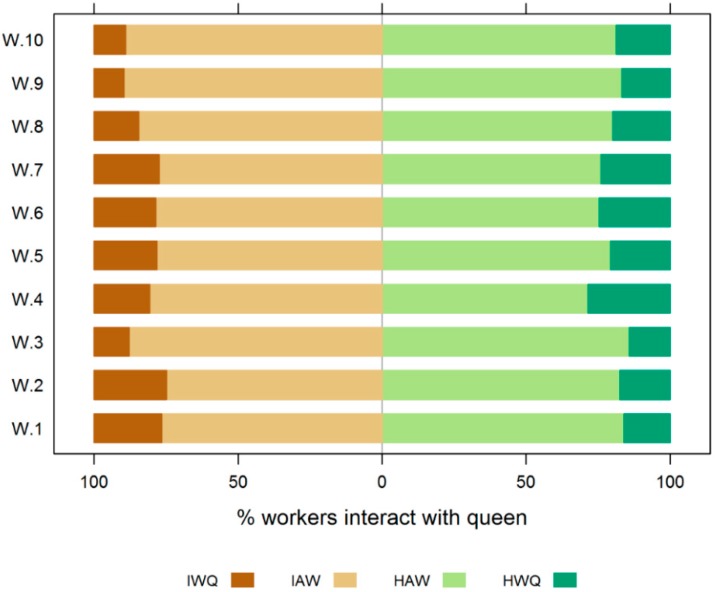
Attendance behavior of infected and healthy workers towards the queen. A small portion of healthy and infected workers stayed close to the screen in contrast to the majority of workers in both groups that did not attend across all trials and days, and there was no significant difference between inoculated and control workers with regards to queen attendance (*p* = 0.320). IWQ: infected workers interact with queen, IAW: infected workers away from queen, HAW: healthy workers away from queen, HWQ: healthy workers interact with queen.

**Figure 6:**
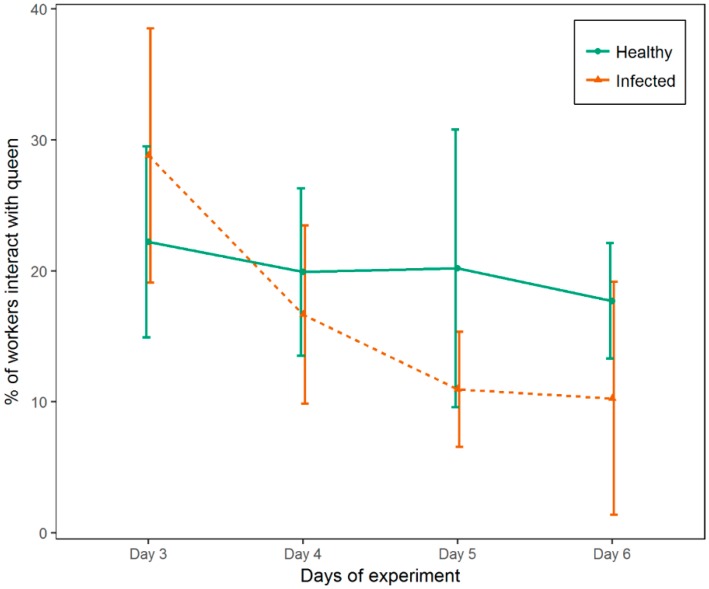
Percentage of honey bee worker attending the queen as a function of the duration of infection. The attendance behavior of infected and control workers changed from a higher proportion of infected workers attending to the queen than healthy workers on experimental Day 3 to the opposite trend on 3 subsequent days, which coincide with the highest mortality rate induced by IAPV.

In both healthy and infected groups of workers, only a small portion of individual workers stay close to the screen, to attend and feed their queen (Figure 5). Overall, no significant difference in attendance behavior was observed between inoculated and control workers (Wilcoxon signed rank test: *p* = 0.320). However, worker behavior changed over time; while queen attendance by inoculated workers was significantly higher than that of control workers on the first day (Fisher’s exact test; *p*_day3_ < 0.001), on all subsequent dates the opposite was true (Fisher’s exact test; *p*_day4_ = 0.007, *p*_day5_ < 0.001, *p*_day6_ < 0.001) (Figure 6).

## 4. Discussion

Honey bee viruses, including IAPV, are known factors in the recent increases of the honey bee colony mortality. Even though honey bee queens are predicted to be particularly well protected, they can be infected with several viruses [14,15,16,49], which may partly explain increased losses and supersedure of queens in managed beekeeping [14,50]. Our results confirm that IAPV can infect honey bee queens by horizontal transmission from workers, indicating that direct physical contact and trophallaxis are two independent means of horizontal IAPV transmission, and suggest that queen–worker interactions may be modulated by the queen to protect herself from infected workers.

Vectoring by the parasitic *Varroa* mite is an important mode of horizontal transmission of several viruses, including IAPV, in honey bee colonies [25,51], but not for queens. IAPV seems to be capable of a number of other transmission routes in honey bees, including feeding, feces, sexual transmission, and vertical transmission [24]. Our study provides clear evidence of direct IAPV transmission from infected workers to queens when queens are forced to interact with these workers. The predominant mechanism of this transmission may be trophallaxis, the transfer of food between individuals. Group living in large colonies enhances virus transmission among individuals via food sharing by routine transfer of nectar, pollen, and bee bread among the colony members. Since IAPV is prominently found in the digestive system [24], transmission through feces is also likely [52]. Fecal deposition was not readily observed in our cages, as bees typically only defecate in flight. However, it is possible that occasional events were quickly cleaned up, and thus, we cannot dismiss that IAPV re-entered our closed system through the foodborne transmission pathway.

The comparison between the IAPV titers in virgin queens of the first and second experiment suggests that direct physical contact can also contribute to IAPV spread, because some queens in direct physical contact with workers exhibited much higher IAPV titers than queens that were separated from infected workers by a wire mesh. However, the majority of queens in physical contact with infected workers had similar IAPV titers relative to the queens in the second experiment, and two queens were not infected at all. In both experiments, queens and workers had trophallactic contact, which was presumably the main route of IAPV transmission in these experiments. Without direct behavioral observations in these experiments, the explanation of these results must remain speculative. A plausible explanation of higher variability of queen IAPV titers in experiment one is that these queens had direct access to food and workers. Thus, some queens may have been able to avoid worker contact altogether, and remained healthy, while others may have had a lot of contact with their infected workers, leading to very high IAPV titers. This interpretation is supported by the three discrete infection categories in queens, in contrast to the more continuous distribution of worker infection levels in the first experiment, and the lack of significant queen infections in the third experiment.

The third experiment showed that mated queens that are provided a choice between IAPV-infected and healthy workers experience a much lower risk of IAPV infection than queens forced to be in contact with infected workers. Only two of the ten queens exhibited IAPV infection, and their infection level was very low. As it was previously shown, queens with opportunity to receive food from healthy workers are less infected by chronic bee paralysis virus [42]. In contrast to the clear consequence for queen infection, neither queen nor worker behavior showed an absolute avoidance of interaction between queens and infected workers. The activity of an individual’s immune system can be perceived by conspecifics in honey bees [40], and IAPV infection evokes strong physiological responses [27,28]. Therefore, it is likely that individuals are able to discriminate between healthy and IAPV-infected individuals. However, it is possible that the wire mesh screen prevented long-range discrimination and impeded most direct contact, weakening the queen’s preference for the healthy worker side in the dual choice design. Our observation could not distinguish contact and trophallaxis, but it is possible that the queens only accepted food from healthy workers and refused food from IAPV-infected workers even when they were associated with the infected workers for a considerable amount of time.

The workers, in turn, did not alter their attendance behavior toward the queen as a consequence of their IAPV infection. Sick workers readily cease social interactions and leave their hive [33], and they are predicted to particularly stay away from the queen. Our overall data did not support this prediction, which may be explained by workers not perceiving the IAPV until the very late stages of the disease. This explanation is supported by the time trend of decreasing proportion of the IAPV-infected workers associating with the queen. Alternatively, relatively healthy workers among the inoculated worker groups may have been the ones associating with the queen, and that proportion becomes smaller over the experimental time period. Based on our pooled quantification of IAPV titers in the workers and lacking individual worker identification, it is impossible to distinguish these two possibilities. However, our overall negative result may also be due to the artificial experimental setup and the small number of workers involved, which may disrupt natural behavior.

Consistently across all three experiments, the queens on average exhibited lower IAPV titers than the inoculated workers. The queen, as the essential individual in the colony and key to vertical disease transmission, should be particularly well defended against infection, but no systematic studies comparing worker and queen immunity in honey bees have been performed. Queens are better protected against oxidative stress [53,54], and a combination of individual traits and social influences affords them an exceptional longevity [8]. The lower IAPV titers observed in these experiments may suggest that queens are better defended by an individual immune system than workers, in addition to social immune mechanisms. However, our observations may also be explained by a difference in the effectiveness of the topical infection (of the workers) versus the oral infection (of the queens). Less time between IAPV infection and sampling in the queens compared to the workers might have also been a contributing factor, although IAPV infections are typically quick to establish, and kill individual bees within a few days if the infection is lethal [23].

## 5. Conclusions

In conclusion, active queen avoidance of accepting food from infected workers may be an underappreciated aspect of social immunity of the honey bee colony, given the crucial role of the honey bee queen for colony function and disease transmission [13]. The experiments described here demonstrate that queens who can feed themselves in the presence of IAPV-infected workers exhibit lower infection rates than queens that must be fed by infected workers, and that the queens who have a choice of being fed by infected and uninfected workers exhibit a lower rate of infection than those that can only be fed by infected workers. The results support the importance of social immunity in colony health and the role of trophallaxis in viral transmission. However, more detailed studies with individually marked bees and continuous monitoring of disease status is needed to better elucidate the social impact of infection on honey bee behavior.

## Supplementary Materials

The following are available online at http://www.mdpi.com/2075-4450/10/1/9/s1, The raw data are deposited and available in the supplementary file.
