# Correction: Amiri, E. et al. Israeli Acute Paralysis Virus: Honey Bee Queen–Worker Interaction and Potential Virus Transmission Pathways. *Insects* 2019, *10*, 9

**DOI:** 10.3390/insects10050123

**Published:** 2019-04-28

**Authors:** Esmaeil Amiri, Gregory Seddon, Wendy Zuluaga Smith, Micheline K. Strand, David R. Tarpy, Olav Rueppell

**Affiliations:** 1 Department of Biology, University of North Carolina at Greensboro, Greensboro, NC 27402-6170, USA; gregvbs@gmail.com (G.S.); zuluagaw1@gmail.com (W.Z.S.); o_ruppel@uncg.edu (O.R.); 2 Department of Entomology & Plant Pathology, North Carolina State University, Raleigh, NC 27695-7613, USA; david_tarpy@ncsu.edu; 3 Life Science Division, U.S. Army Research Office, Research Triangle Park, Durham, NC 27709-2211, USA; micheline.k.strand.civ@mail.mil

It has been brought to our attention that one note was missing in the Funding section of our published paper [1]. 

**Funding**: This research was supported by the U.S. Army Research Office (Cooperative Agreement: W911NF1520045) and USDA-APHIS (agreements #17-8130-0636-CA and #18-8130-0636-CA) and performed while Esmaeil Amiri held an NRC Research Associateship award at the UNCG Social Insect Lab.

The authors would like to note that this addendum does not cause any changes to the results and conclusions in the original published paper.
